# Influence of Variation in Hind Leg Structure of Auchenorrhyncha on Their Jumping Performance

**DOI:** 10.3390/biology14040418

**Published:** 2025-04-13

**Authors:** Yifei Xu, Christopher H. Dietrich, Wu Dai

**Affiliations:** 1 Key Laboratory of Plant Protection Resources and Pest Management, Ministry of Education, College of Plant Protection, Northwest A&F University, Yangling 712100, China; xyf@cxtc.edu.cn; 2 Illinois Natural History Survey, Prairie Research Institute, University of Illinois, Champaign, IL 61820, USA; chdietri@illinois.edu

**Keywords:** SEM, structure of hind legs, high-speed capture, locomotion, Auchenorrhyncha

## Abstract

Four species representing four different families of the hemipteran insect suborder Auchenorrhyncha, *Lepyronia coleoptrata* (Aphrophoridae), *Euricania ocellus* (Ricaniidae), *Kolla* sp. (Cicadellidae) and *Tricentrus* sp. (Membracidae) were investigated using high-speed photography and scanning electron microscopy to identify hind leg structures that may influence jumping performance. The coxa–trochanteral joint, femur and tibia were found to have distinct structural adaptations that vary among these jumping insects. Froghoppers and planthoppers possess a coxal protrusion which is absent in leafhoppers and treehoppers, the latter featuring a more recessed coxal fossa. The medial coxae of these insects exhibit fields of microtrichia that vary in density and fine structure. Medial gears on the trochanters of *Tricentrus* sp. are implicated in the storage of energy prior to their jumps. These structural differences manifest in the insects’ jumping performance. The study demonstrated a correlation between the robustness of the microtrichia field interaction and the insect’s jumping capability. Specifically, leafhoppers, equipped with a pair of rivet-like structures connecting the hind coxae, were observed to achieve quicker and more stable take-offs. The study reveals that structural variations in the hind legs of Auchenorrhyncha species significantly influence their jumping performance, with implications for both efficiency and stability.

## Simple Summary

Although small insects generally have similar jumping mechanisms, their jumping performances exhibit significant variations. By conducting a comparative analysis of the external morphology of the hind legs using scanning electron microscopy (SEM) and recording jumping motions using high-speed capture technology, this study quantitatively evaluated the physical parameters generated during the jumping process. The results indicate that differences in the morphological structure of the hind legs significantly affect jumping performance, specifically manifested as notable divergence in key metrics, such as jumping height, distance, and initial velocity among different species. This finding provides important experimental evidence for the further understanding of the biomechanical mechanisms of insect jumping behavior.

## 1. Introduction

Insects renowned for their extraordinary jumping abilities have evolved intricate morphological adaptations across their locomotor systems, including specialized limbs, joints, muscles and movement patterns, with particular specialization in their propulsive hind legs. The evolutionary development of jumping mechanisms across insect taxa reflects selective pressures for predator evasion through enhanced acceleration capabilities and biomechanical optimization of jump performance. Biomechanically, leg-driven jumping insects are categorized by their primary torque-generating joints [1,2]: (1) The Femur–Tibia (FT) mechanism utilizes energy storage and rapid joint extension [3], enabling swift movement through fast muscle contraction, as observed in locusts [4]; (2) The Trochanter-driven system (exemplified by froghoppers, planthoppers, leafhoppers, treehoppers, fleas) employs pleural arch spring recoil to generate torque at the coxa–trochanter joint (PADT mechanism: Pleural Arch Depression of the Trochanter) [5,6]. While some orthopterans, like crickets, achieve jumping through direct muscular torque [7], most high-performance jumpers supplement muscle action with exoskeletal elastic energy storage through catapult mechanisms. These systems combine specialized cuticular structures with mechanical advantage from elongated legs, achieving power outputs exceeding direct muscle capacity by three orders of magnitude [8,9,10,11,12,13,14], as demonstrated in grasshoppers [4], flea beetles [10], and all PADT jumpers. The rapid release of stored elastic energy produces greater propulsive forces and accelerations than muscular action alone [11,12], achieving remarkable velocities within millisecond timescales through energy release rates surpassing typical muscle contraction limits by 1000-fold [13,14].

Auchenorrhyncha (Insecta: Hemiptera), comprising Cercopoidea, Fulgoroidea, Membracidae and Cicadellidae, represent the pinnacle of insect jumping performance through extraordinary morphological adaptations [15,16]. Kinematic analyses reveal a conserved jumping sequence across these lineages: initial synchronized hind leg retraction positions the tibiae medially under the body, followed by simultaneous femur–tibia extension and coxa–trochanter depression [17,18]. Notably, while sharing this fundamental jumping mechanism, substantial performance divergences exist among families, correlating with ultrastructural specialization of metathoracic appendages. Planthoppers and froghoppers employ an elastomeric catapult mechanism through thoracic skeletal composites comprising resilin-rich elastic pads sandwiched between sclerotized cuticular layers [19,20]. This system features a coxal protrusion engaging with specific femoral regions for energy storage, complemented by microtrichia fields on coxal surfaces providing traction through cross–mesh interactions [17,19]. In contrast, leafhoppers (Cicadellidae), lacking both the energy-storing protrusions and resilin-containing coxal structures, achieve comparable performance through a unique evolutionary innovation: intermeshing microtrichia field with embedded rivet-like structures within the coxae [21]. This convergence highlights the alternative evolutionary pathways for achieving high-performance jumps.

The structure–function paradigm is further illustrated by specialized hind leg adaptations across diverse taxa, tailored to their environments for efficient locomotion. The Papuan weevil *Trigonopterus oblongus* (Pascoe) demonstrates a functional screw-and-nut system in its coxa–trochanteral joints, enabling substrate anchoring during feeding through chrysomeloid joint modification [22]. Nymphs of *Issus* planthoppers employ meshed gears in their femoro–trochanteral joints to synchronize leg movements with microsecond precision [23]. These biological innovations have inspired biomimetic designs, including froghopper-inspired jumping robots with symmetrical legs and synchronized gear systems [24].

This study investigates the specialized hind leg mechanisms underlying the jumping supremacy of Auchenorrhyncha. We analyze the functional morphology of the coxo–trochanter joint complex and its performance implications, with particular focus on a novel femur–coxa locking mechanism that enables sequential energy storage through gradual muscular tensioning followed by rapid elastic release. This mechanism potentially represents an evolutionary optimization for explosive power generation, providing new insights into the biomechanical diversification of insect jumping systems.

## 2. Materials and Methods

Adult specimens of *Lepyronia coleoptrata* (Linnaeus, 1758), *Euricania ocellus* (Walker, 1851), *Kolla* sp. and *Tricentrus* sp. were collected from low-growing shrubs in Yangling, Shaanxi Province. China (34°27′ N, 108°9′ E, elev440m). From June to September each year between 2021 and 2023, the numbers for each group were as follows: 17, 13, 14, and 9, respectively. Specimens were preserved in 95% ethanol and stored at 4 °C. The adult samples studied included both females and males.

### 2.1. Samples for SEM

Adult specimens were initially immersed in 95% ethanol. Their legs were excised using dissecting needles under a stereomicroscope (Olympus SZX10, Tokyo, Japan) and then dehydrated in 100% ethanol for two intervals of 30 min each. Subsequently, they were gradually transitioned through a series of tert-butyl ethanol (TBA) solutions at concentrations of 25%, 50%, and 75%, with ethanol to TBA ratios of 3:1, 1:1 and 1:3, respectively, for 15 min per concentration, followed by a final immersion in 100% TBA for 30 min. Dehydrated samples were then freeze-dried (VFD-21S, SHINKKU VD, Tokyo, Japan) for 3 h. The dried specimens were mounted on aluminum stubs with double-sided copper sticky tape and sputter-coated with a 40/60 gold/palladium alloy using a high-resolution coater (MSP-1S, SHINKKU VD, Tokyo, Japan). The SEM observations were conducted employing a T-3400 SEM (Hitachi, Tokyo, Japan) at an operating voltage of 15 kV or a Nova Nano SEM-450 (FEI, Hillsboro, OR, USA) at 5–10 kV.

### 2.2. High-Speed Photography

Sequential images of the insects’ jumps were captured using a high-speed camera at a frame rate of 10,000 $s^{-1}$ with an exposure time of 1 μs. The imaging system utilized was the X213 model (Revealer, Hefei, China), equipped with a 105 mm micro lens.

Jumps were either spontaneous or induced by gentle mechanical stimulation using a fine paintbrush within a fully enclosed room that allowed insects to jump freely. Due to the limited capture field of the camera, there was a risk of insects exiting the frame and insects were placed on a 10 mm × 10 mm × 30 mm cube platform, the surface of which was wrapped with a wet paper towel to provide traction for the insects at take-off. The high-speed camera was positioned to capture the side view of the jumping process, focusing on the edge of the platform. The body angle was defined as the angle formed by the longitudinal body axis in relation to the horizontal plane. The peak velocity during take-off was calculated by tracking a selected mass point on the insect’s body using the processing software of the high-speed camera. The first movement of the hind legs occurred at the capture moment point. The moment when the hind legs lost contact with the ground, marking the insect as airborne, was designated as time t = 0 ms. This reference point allowed for the alignment and comparison of different jumps. The initial movement of the hind leg was marked out, and the time elapsed between this movement and take-off was used to calculate the acceleration of the body during the jump. The tergum of *Kolla* sp. was affixed to a transparent glass background using adhesive tape. A high-speed camera positioned vertically below the insects captured the interaction of their hind legs during the jump, providing footage of the ventral surface movement.

### 2.3. Image Processing and Measurement

The imported photographs were observed and measured utilizing Adobe Photoshop CS6 (Adobe Systems, San Jose, CA, USA). All characters are expressed as mean ± SEM. Statistical analyses were conducted using SPSS 19.0 (SPSS, Chicago, IL, USA). Graphs were produced using Microsoft Office Excel 2007.

Body length measurements were obtained from scanning electron microscopy (SEM) images using Photoshop CS6 software (Adobe Systems, San Jose, CA, USA). Adult insect mass was quantified immediately post-mortem using an analytical balance (LS220ASCS, Precisa, Zurich, Switzerland), following high-speed video recordings of their jumping behavior.

## 3. Results

### 3.1. Morphological Structure

Significant variations in body shape were observed among the insects analyzed, despite similar body lengths, accompanied by a 5-fold variation in body mass, ranging from 3.9 to 18.6 mg (Table 1). The tibia, being the longest segment on the insects’ hind legs, plays a crucial role in their jumping ability. The spindly hind legs of these insects enable even the smallest species to achieve superior jumping performance. Moreover, different structural features of these legs also influence their jumping capability and stability.

**Figure 1:**
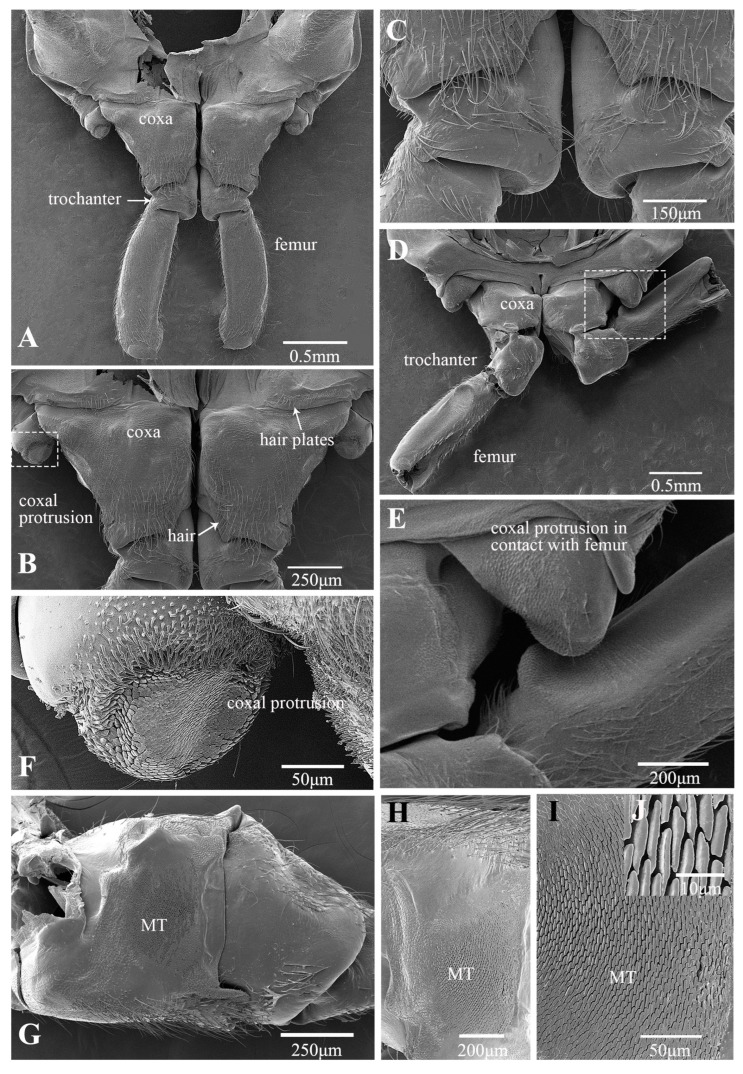
SEM of the proximal hind leg joints of *Lepyronia coleoptrata*: (**A**) dorsal view of coxae and trochanters; (**B**) enlarged view of (**A**); (**C**) setae on coxo–trochanteral joints; (**D**) ventral view of coxae and trochanters; (**E**) enlarged view of white outlined box in (**D**), showing coxal protrusion in contact with the specific location on the femur; (**F**) coxal protrusion; (**G**) medial view of coxa and trochanter; (**H**) enlarged view of (**G**), showing Microtrichia (MT); (**I**,**J**) enlarged view of Microtrichia (MT).

**Figure 2:**
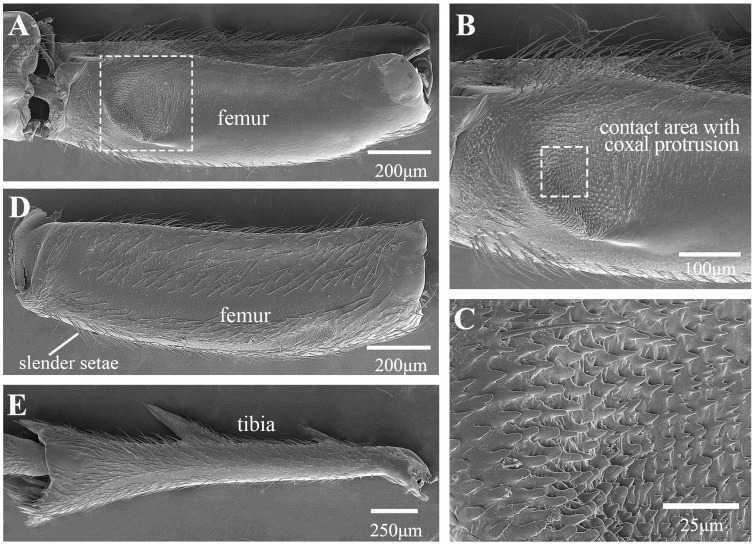
SEM of femur and tibia of *Lepyronia coleoptrata*: (**A**) depression on ventral surface of femur; (**B**) enlarged view of white outlined box in (**A**), showing contact area with coxal protrusion; (**C**) enlarged view of white outlined box in (**B**), microtrichia on the femur protrusion; (**D**) dorsal view of femur; (**E**) whole tibia showing spines.

In the froghopper, *Lepyronia coleoptrata*, the folded forewings extend posteriorly to cover most of the hind legs when viewed from the side. The tibia is slightly more than twice as long as the femur and the total length of the hind leg is 84% of the total body length (Table 1). The coxae and trochanters, along with the interface between the trochantin and prosternum, are adorned with setae (Figure 1A–C). Notably, the posterior section of the coxa, which connects to the trochanter, features a concentration of longer setae (Figure 1C). The broad coxae have prominent bulbous protrusions on their lateral sides (Figure 1B). Spike-like microtrichia on protrusions are grouped into an oval, domed-shaped structure in the middle, with height gradually decreasing from the periphery to the center until it forms a smooth plane (Figure 1F). When the leg is retracted prior to jumping, this structure is in contact with a specific location on the femur of the hind legs (Figure 1D,E). A microtrichia field on the inner (medial) side of the coxae, where the coxae meet closely (Figure 1G), features microtrichia with raised edges and a depressed middle, forming a sucker shape (Figure 1G–I). The ventral surface of the femur is flat and smooth, except at the apex, where there is a depression significantly lower than the surrounding area oriented toward the connection with the coxal protrusion (Figure 2A) and featuring spiral inwardly oriented spikes that eventually become horizontally lamellar (Figure 2B). When the hind leg is retracted and elevated prior to jumping, the depression on the femur is pressed against the coxal protrusion (Figure 1D and Figure 2C). This action brings the microtrichia on the two surfaces directly into contact with each other (Figure 1D and Figure 2C). The femurs are relatively stout, bearing a few rows of slender setae on the dorsal side (Figure 2D). The two large spines protruding laterally from the tibia are an important distinguishing feature of this species and some other froghoppers, but their function remains to be determined. A row of stiff spines is present ventrally at the apex of the tibia adjacent to its junction with the tarsi (Figure 2E).

**Figure 3:**
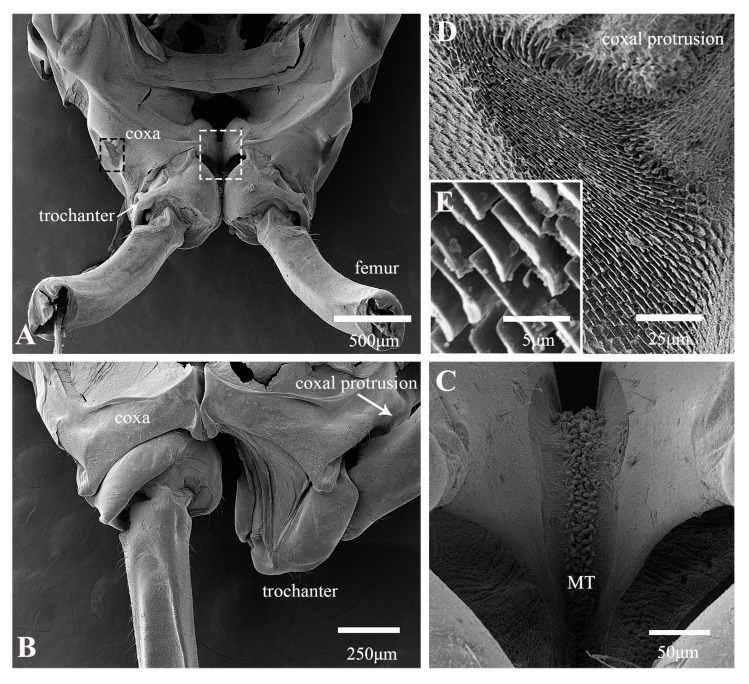
SEM of the proximal hind leg joints of *Euricania ocellus*: (**A**) dorsal view of coxae and trochanters; (**B**) ventral view of coxae and trochanters; (**C**) enlarged view of white outlined box in (**A**), showing interlocked microtrichia (MF) fields of coxae; (**D**) enlarged view of black outlined box in (**A**), showing field of lamellar microtrichia; (**E**) enlarged view of lamellar microtrichia.

**Figure 4:**
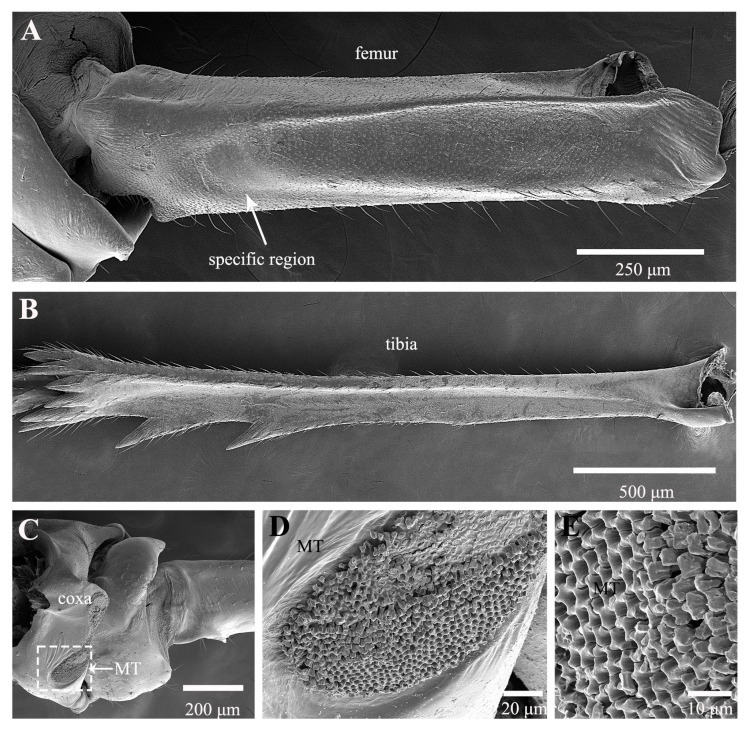
SEM of hind femur and tibia of *Euricania ocellus*: (**A**) ventral view of femur showing sunken part contacted and depressed with coxal protrusion; (**B**) tibia showing lateral spines; (**C**) medial aspect of the coxa with the microtrichia field; (**D**) enlarged view of white outlined box in (**C**), showing the microtrichia field covered in wavy microtrichia; (**E**) enlarged view of microtrichia.

The forewings of ricaniid planthoppers are broadly triangular and are held tent-like over the body when at rest, forming a distinctive ridge along the dorsal body surface. In *Euricania ocellus*, the hind legs span an impressive 86% of the body length (Table 1). The tibia is only about 1.5× the length of the femur. The coxae of the two legs are closely adjacent, yet the trochanters possess the remarkable ability to rotate outward in preparation for a jump (Figure 3A,B). The rotation elevates the femora upward until they make contact with the coxal protrusions, which are densely covered with a lamellar array of microtrichia (Figure 3D,E). This critical juncture is not only where the hind leg can perform its lift and squeeze motion but also serves as the pivotal point for initiating a jump, contingent upon engagement with a specific region of the femur (Figure 4A). The medial surfaces of the coxae together form an hourglass-shaped structure (Figure 3C). Wavy structures project from the surface, intertwining in an irregular and complex manner and, thus, increasing the area of contact (Figure 4C–E). This unique architecture helps synchronize the movement of the hind legs to a certain degree (Figure 3C). It ensures the stability of the jump and is instrumental in storing a portion of the energy required. The tibiae are slender and robust, equipped laterally with two large spines, and also have a cluster of multiple spines at their junction with the tarsi (Figure 4B).

**Figure 5:**
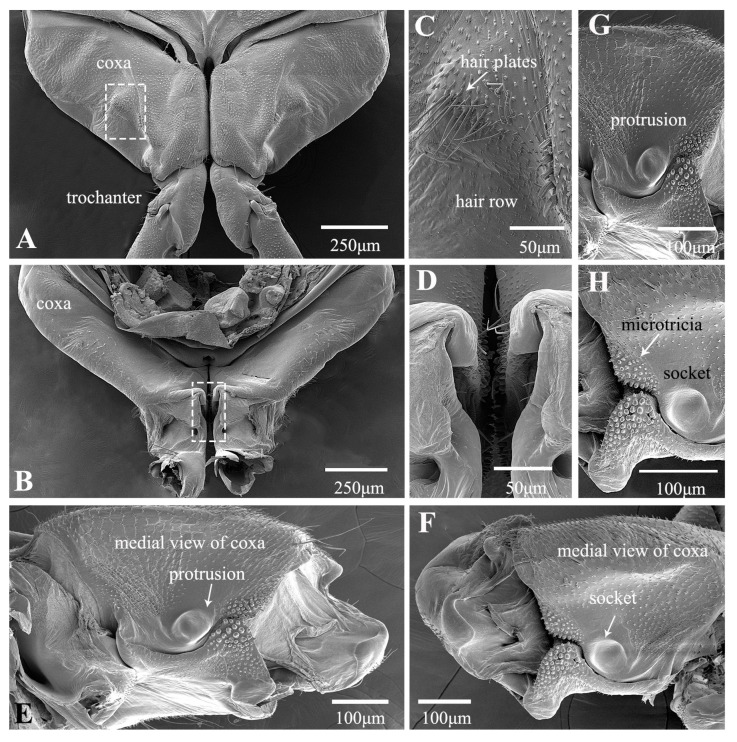
SEM of the proximal hind leg joints of *Kolla* sp.: (**A**) dorsal view of coxae and trochanters; (**B**) ventral view of coxae and trochanters; (**C**) enlarged view of white outlined box in (**A**), showing hair plate and hair row; (**D**) enlarged view of white outlined box in (**B**), showing interlocked microtrichia fields of coxae; (**E**) medial aspect of the coxa, showing protrusion; (**F**) medial aspect of the coxa, showing socket; (**G**) enlarged view of the coxa from medial view, showing protrusion; (**H**) enlarged view of the coxa from medial view, showing socket and microtrichia.

**Figure 6:**
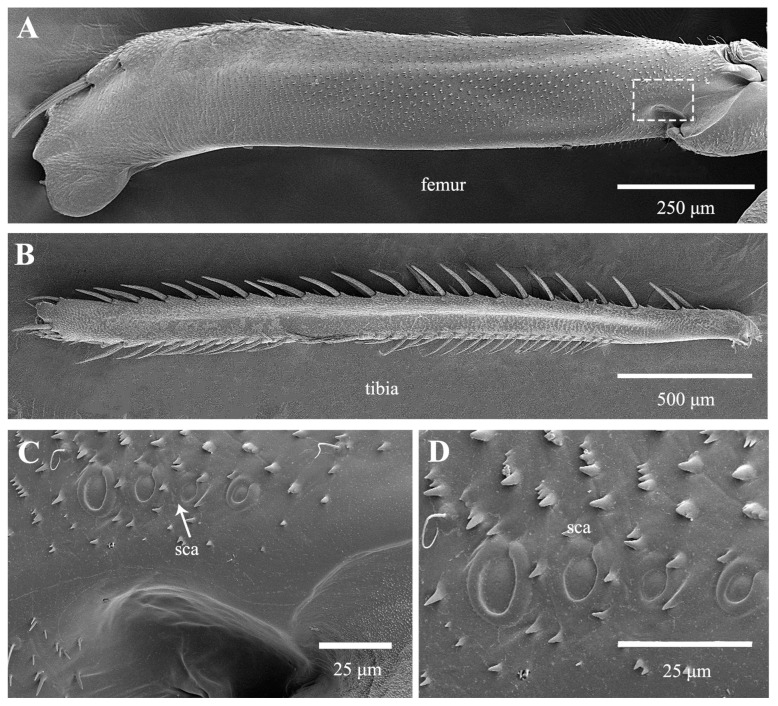
SEM of femur and tibia of *Kolla* sp.: (**A**) dorsal view of femur; (**B**) spines of the tibia; (**C**) enlarged view of white outlined box in (**A**), showing sensilla campaniformia (Sca); (**D**) enlarged view of sensilla campaniformia (Sca).

The leafhopper *Kolla* sp., possesses a uniform and smooth silhouette, marked by a streamlined form that presumably optimizes its aerodynamic efficiency. Although lighter in body mass compared to the studied froghopper, planthopper and treehopper, the hind legs are exceptionally long, constituting 88% of total body length (Table 1). The coxae have a dorsal depression that accommodates the femur just before a jump (Figure 5A). Within this depression lies a cluster of long hair plates, while a few shorter, thicker setae are situated on the side of the protrusion, adjacent to the hair plates (Figure 5C). Prior to jumping, the femora are lifted forward until they rest in these depressions, where they are squeezed against the hair plates and rows. The coxae of the two hind legs are intimately connected, and when pried open along the midline, they are seen to be linked by a protrusion from the medial wall of one coxa that fits into a socket of the other coxa (Figure 5B,D–H). This structure, in both shape and function, resembles a rivet. Two arrays of microtrichia adjacent to the protrusion and socket naturally engage with each other when the coxae are interlocked (Figure 5H). The femora are elongated and gradually taper from their junction with the tibia to the trochanters (Figure 6A). A row of four sensilla campaniformia is located near the trochanters (Figure 6C,D). The longitudinal rows of spine-like macrosetae on the tibia are the most distinctive features of leafhoppers (Figure 6B). Only a few relatively small spines are present at the apex of the tibia near its junction with the tarsi.

**Figure 7:**
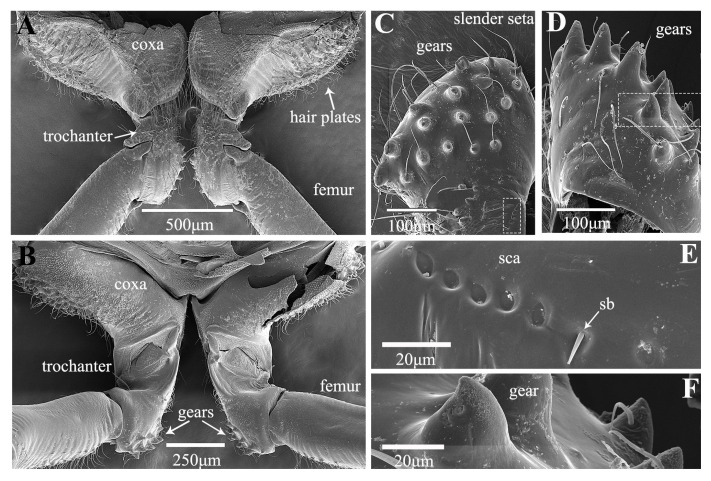
SEM of the proximal hind leg joints of *Tricentrus* sp.: (**A**) dorsal view of coxae and trochanters, showing hair plates; (**B**) ventral view of coxae and trochanters, showing gears; (**C**) medial view of gears, showing slender setae; (**D**) lateral view of gears; (**E**) enlarged view of white outlined box in (**C**), showing sensilla campaniformia (Sca) and sensilla basiconica (Sb); (**F**) enlarged view of gear.

**Figure 8:**
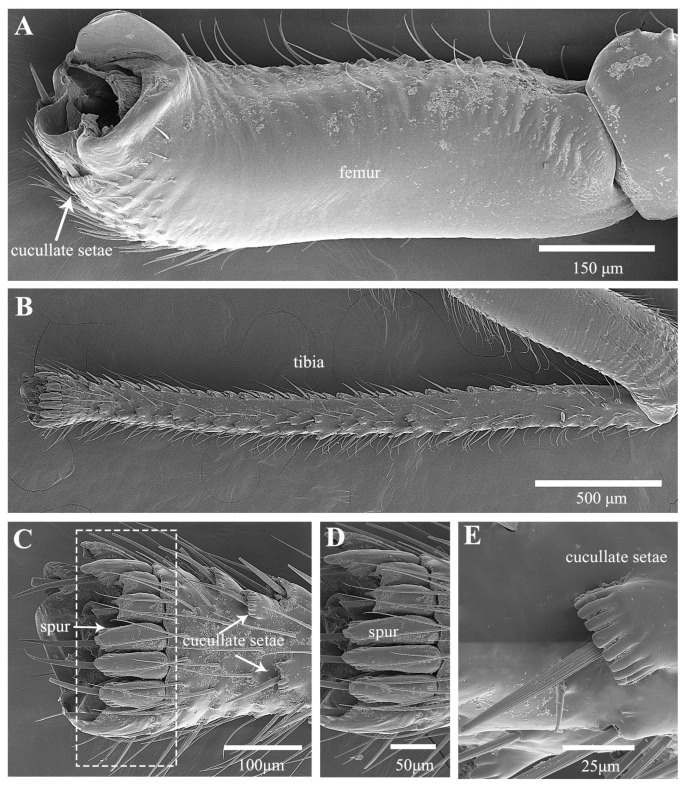
SEM of femur and tibia of *Tricentrus* sp.: (**A**) ventral view of femur; (**B**) tibia; (**C**) apex of tibia; (**D**) enlarged view of outlined box in (**C**), showing spurs; (**E**) cucullate seta.

Treehoppers are noteworthy for their enlarged and often highly modified pronotum, but their hind leg structure is similar to that of leafhoppers. In *Tricentrus* sp., the hind legs are notably long, constituting 85% of the body length (Table 1). When viewed dorsally, the coxae resemble those of the leafhopper, featuring a concave surface equipped with hair plates (Figure 7A,B). The trochanters of *Tricentrus* sp. possess an array of gears with curved tips that point toward the coxa. These gears each bear a slender apical seta (Figure 7C,D,F). Adjacent to the gears, a row of six sensilla campaniformia (Sca) and a sensillum basiconicum (Sb) are present (Figure 7E), serving as crucial mechanical sensors that monitor leg movements. The ventral aspect of the femur is smooth and has only a few fine setae (Figure 8A). The distal end connecting with the tibia is adorned with several cucullate setae (Figure 8A,E). The tibia is slender, with longitudinal rows of cucullate setae, and terminates in an enlarged end equipped with a transverse row of spurs (Figure 8B–E).

### 3.2. Kinematics of the Jump

**Figure 9:**
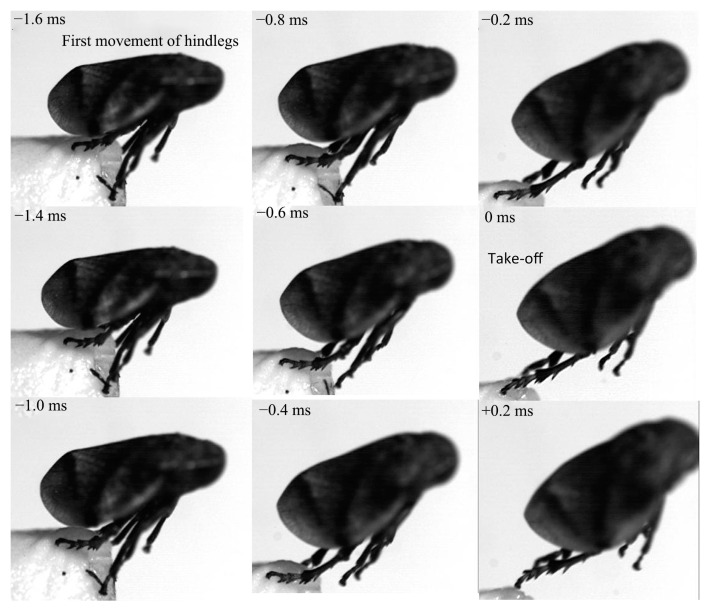
Side view of *Lepyronia coleoptrata* jumping from a horizontal surface, captured at 10,000 images $s^{-1}$ and exposure time of 1 μs, take-off was designated as t = 0 ms.

**Figure 10:**
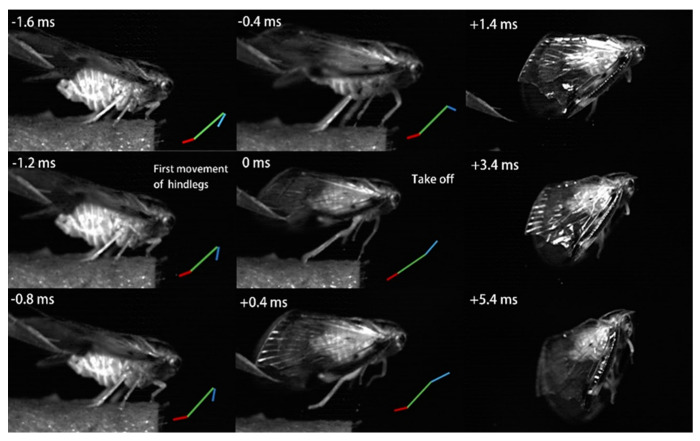
Side view of *Euricania ocellus* jumping from a horizontal surface, captured at 10,000 images $s^{-1}$ and exposure time of 1 μs, take-off was designated as t = 0 ms. Positions of the different segments of the right hind leg are indicated by color-coded lines: femur in blue, tibia in green and tarsus in red.

**Figure 11:**
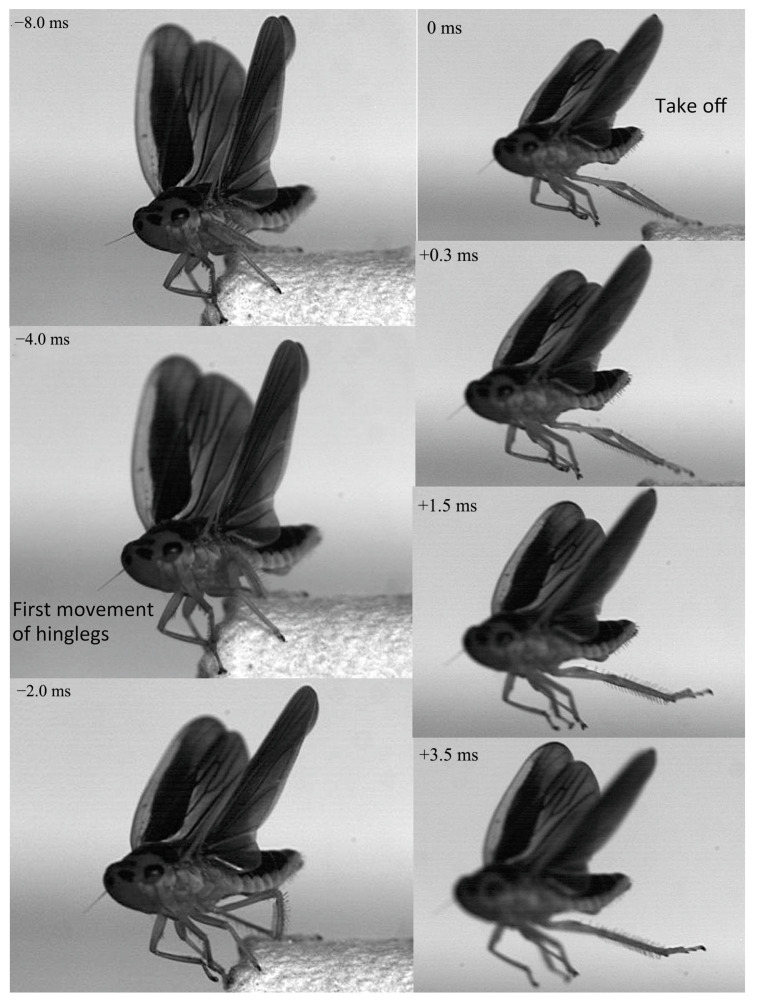
Side view of *Kolla* sp. jumping from a horizontal surface, captured at 10,000 images $s^{-1}$ and exposure time of 1 μs. Take-off was achieved in 0 ms.

**Figure 12:**
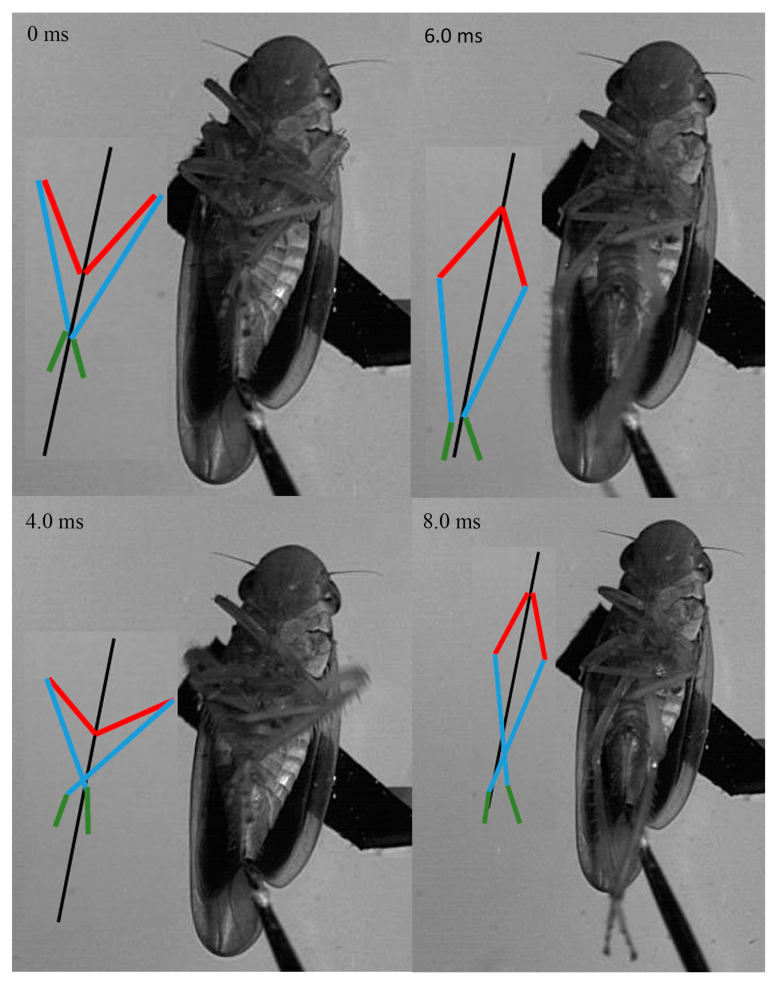
Ventral view of jumping motion in *Kolla* sp., captured at 10,000 images $s^{-1}$ and exposure time of 1 μs. The positions of the different segments of the right and left hind legs are indicated by the color-coded lines: femur in red, tibia in blue and tarsus in green, with the midline body axis in black.

**Figure 13:**
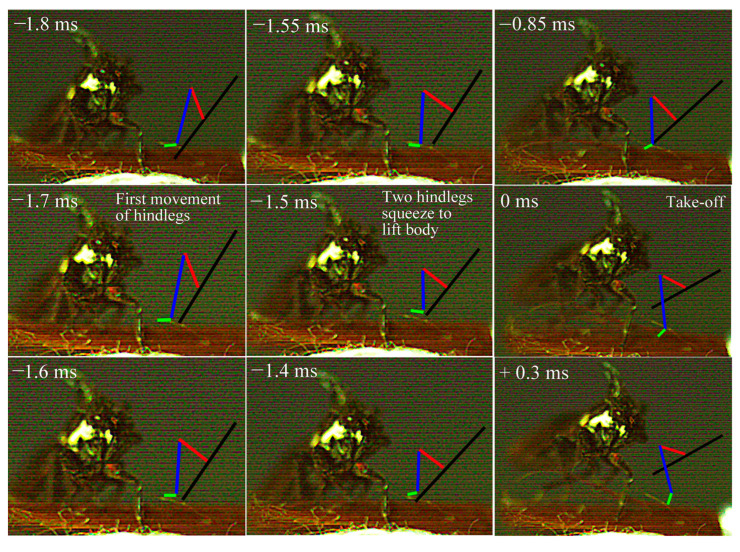
Side view of *Tricentrus* sp. jumping from a horizontal surface, captured at 10,000 images $s^{-1}$ and exposure time of 1 μs. Take-off was achieved in 1.7 ms. The positions of the different segments of the right and left hindlegs are indicated by the color-coded lines: femur in red, tibia in blue and tarsus in green, with the midline body axis in black.

**Figure 14:**
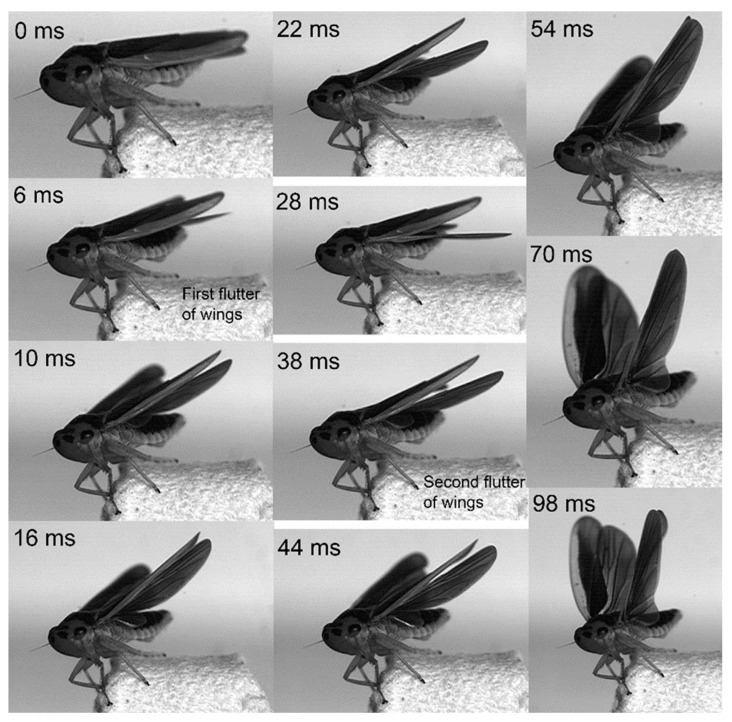
Side view of *Kolla* sp. flapping wings before jump, captured at 10,000 images $s^{-1}$ and exposure time of 1 μs.

Jumping movements of the four species were analyzed using high-speed video recordings captured from a lateral view (Figure 9, Figure 10, Figure 11, Figure 12, Figure 13 and Figure 14). The side views of insects jumping in the horizontal plane provided detailed insights into the movement of the different legs, particularly the initial movement of the hind leg and the moment at which contact with the surface was lost (Figure 9, Figure 10, Figure 11 and Figure 13). A ventral view of the jumping process of the leafhopper clearly illustrated the motion of each hind leg segment and the synergy in movement between the two hind legs (Figure 12).

The initial movement of the hind legs in the jumping preparation phase involves pressing down on the body and simultaneously rotating the coxa–trochanteral joints to lift. This action facilitates the forward rotation of the trochanters and the extrusion of the femora towards the coxae to make contact with the coxal protrusion, which is crucial for triggering the jump. As the femora are rotated forward, the tibiae follow suit, positioning themselves alongside the body with the tarsi in contact with the surface (Figure 9, Figure 10, Figure 11 and Figure 13). This preparatory motion is usually sustained for a brief period, allowing for adjustment in the positions of the front and middle legs and elevating the body angle relative to the substrate. The take-off angle (relative to horizontal) varied as follows: 37° in *Lepyronia coleoptrata*, 48° in *Euricania ocellus*, 30° in *Kolla* sp. and 29° in *Tricentrus* sp. (Table 2).

Following the preparation period, a swift downward press and extension of the hind legs marks the initiation of the jump, propelling the body forward in a rapid take-off. The initial phase of this backward movement is identifiable by the depression of the coxa–trochanteral joint, best observed from the ventral view (Figure 11). When viewed from the side, the coxa–trochanteral joint is partially obscured by the wing; however, the first movement discernible is of the femur, connected to the tibia, moving downward and backward, which causes the tarsus to maintain contact with the ground. This coordinated motion quickly thrusts the body forward and upward, lifting it off the surface. The middle leg is the first to lose contact with the surface, followed by the front leg. In the jump’s final stage, only the pretarsus of the hind leg remains in contact with the surface, providing the last burst of propulsion (Figure 11). Throughout the jump, the body pushes forward, its speed increasing. At the moment of take-off, when the hind leg leaves the ground, the body’s speed peaks. Once airborne, the speed gradually decreases due to air resistance. At or immediately after take-off, the tarsi of the two hind legs converge at the body’s midline and, in some instances, cross each other. During the initial flight trajectory of the jump, the hind legs remain fully extended downward (Figure 12).

### 3.3. Motion Physics

Although the four species studied had similar hind leg movements during the preparatory phase for take-off, their trajectories after take-off varied significantly. All four species are comparable in body length, but *Lepyronia coleoptrata* is the heaviest, with a mass five times that of *Kolla* sp. This substantial body weight somewhat hinders its jumping capability, resulting in a maximum velocity of 2 $ms^{-1}$ (Table 2). *Lepyronia coleoptrata* tends to tilt and rotate to one side upon take-off, which impedes smooth flight and often impedes a smooth landing. In contrast, *Euricania ocellus*, which has a similarly broad thorax, takes less time (1.2 ms) in the preparatory stage before jumping and can achieve a maximum speed of 2.8 ${ms}^{-1}$ (Table 2). Its aerial trajectory is smoother, with greater vertical acceleration. *Kolla* sp., the lightest of the species studied, has the longest jumping preparation phase (t = 4 ms) and can reach the highest take-off speed (v = 3.9 $ms^{-1}$) (Table 2). Its jumping trajectory closely resembles an ideal smooth parabolic arc. *Tricentrus* sp., the smallest of the four species, shares this superior jumping ability, but its jumping performance is erratic, with the body often deflecting to one side and tumbling. The energy expenditure of the four species’ jumps ranged from 24 to 45 μJ, with a power output of 16 to 38 mW and exerted forces between 3 and 27 mN.

In the jumps previously described, the wings remained folded, indicating that they did not contribute to jumping performance. However, in certain instances, the wings were extended and flapped prior to take-off, facilitating a smooth transition into sustained winged flight (Figure 14). It is noteworthy that, in cases when the wings were extended prior to a jump, the timing of take-off, marked by the moment the hind legs left the surface, varied depending on the phase (ascent and descent) of the wing flapping cycle. In the above examples, neither *Lepyronia coleoptrata* nor *Euricania ocellus* exhibited wing flapping during preparation for jumping, at the moment of take-off, or after take-off. In contrast, *Kolla* sp. was observed to flap its wings before take-off. However, in some other taxa, wing flapping may occur during the preparation phase or while ascending.

**Figure 15:**
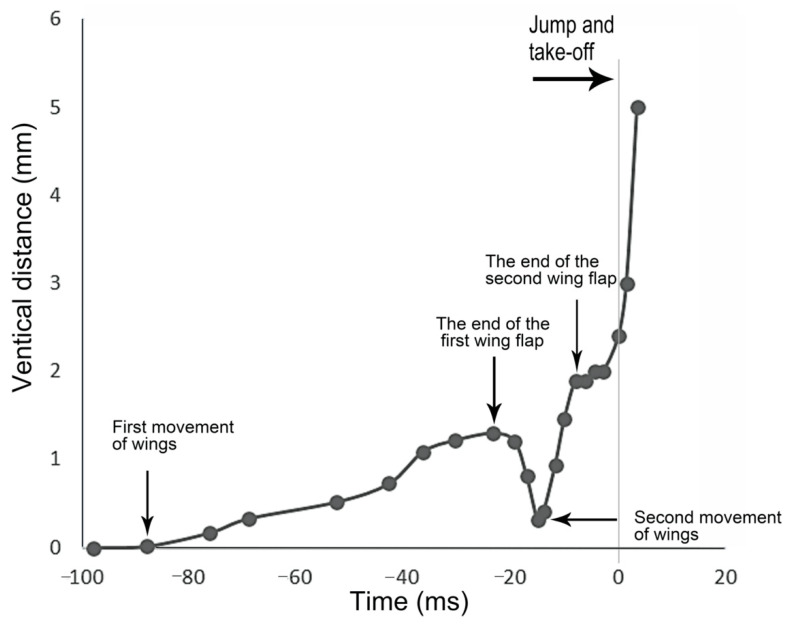
The hind leg and wing movements of *Kolla* sp. during the jump are shown in Figure 14. The vertical distance of the wing flapping action relative to the resting state was recorded for 100 ms before take-off, using the wing resting parallel on the back of the body as the baseline. The gray vertical line represents the moment of take-off.

Wing flapping during take-off occurred over more than 100 ms, with the wings elevated and held in this position for some time, then accompanied by the extension of the hind legs to propel the body forward. This flapping action constituted two distinct cycles, each contributing to the insect’s ascent to a certain height. In the initial cycle, the wings were elevated for an extended period but did not immediately reach their zenith. Subsequently, the wings were swiftly retracted. The second cycle involved a rapid lift of the wings to their highest point, which was maintained for a brief interval before take-off. During this second cycle, the wings were held at their maximum height, creating a period in which any vertical movement was attributable to the hind legs performing the preparatory phase for lift-off (Figure 15).

## 4. Discussion

The present study confirms that Cercopoidea, Fulgoridae, Membracidae and Cicadellidae, despite sharing a common set of jumping mechanisms, have each evolved distinct strategies to enhance their leaping capabilities. Cercopoidea utilize a robust catapult mechanism known as PADT (pleural arch + depressor trochanter), which includes a mechanical locking mechanism, to generate the necessary propulsion force for their jump [25]. Fulgoridae, on the other hand, rely on their hind legs in conjunction with an elastic amplifier to interact with the surface, facilitating the completion of their jump [26]. Membracidae are believed to employ a power amplification mechanism that operates in a catapult-like fashion [27]. Cicadellidae, with proportionately longer hind legs, require a longer take-off time. Despite this, they exhibit remarkable jumping performance, facilitated by the storage of energy during the preparatory phase. Although Cicadellidae exhibit stronger jumping performance compared to Cercopoidea and Fulgoridae, their take-off time is longer, which somewhat reduces their jumping efficiency.

All four studied insects depend on their long, slender hind legs to execute their jumps, with the femur and tibia accounting for the majority of the hind leg’s length. In *Lepyronia coleoptrata*, three distinct arrays of setae on the coxa and trochanter are strategically positioned to serve as proprioceptors, signaling key movements during the jumping process. Prior to a jump, the femur presses to the coxa, making contact with a bulbous coxal protrusion that acts as a trigger for the jump. Surrounding this area, spike-like microtrichia form an oval, domed-shaped structure [28]. A substantial field of microtrichia (MT) on the hind coxae increases the contact area, ensuring synergy and a certain interlocking effect that stores energy prior to the jump [29]. *Euricania ocellus*, unlike *Lepyronia coleoptrata*, possesses a coxal protrusion covered with interlaced lamellar microtrichia that interact with the smooth end of the femur, also serving as a trigger for the jump [15,16]. Additionally, the coxae of the hind legs have a field of hourglass-shaped microtrichia on the mesal surfaces, which interlock more effectively than the toothlike microtricha of *Lepyronia coleoptrata*. *Kolla* sp. lacks a coxal protrusion but has hair plates and rows within the coxa’s depression that make contact with the femur. Notably, besides the two fields of pillar-shaped microtrichia on the interior surfaces of the hind coxae, *Kolla* sp. also possesses a pair of interlocking structures on the coxae resembling rivets in shape and function [21,30]. The coxa of *Tricentrus* sp. is similar to that of *Kolla* sp., with a concave surface that contacts the femur. However, unlike the other three species, *Tricentrus* sp. is distinguished by the presence of gears on the trochanters, marking it as the sole species among the four with this feature.

The jumping mechanisms of the insects studied here are similar to those of other previously studied small Auchenorrhyncha species [19,30,31]. Despite the similar body lengths of the four studied species, differences in the hind leg length itself do not significantly explain differences in jumping ability [6]. The increased contact area of the coxal microtrichia of *Lepyronia coleoptrata* resulted in less effective synergism between the hind legs compared to the intermeshed microtrichia of *Euricania ocellus* and *Kolla* sp. This difference may explain why *Lepyronia coleoptrata* tends to lean during take-off and struggles to maintain stable flight. In contrast, the meshing microtrichia in *Euricania ocellus* ensure synchronized hind leg movement during take-off and stability in gliding. *Kolla* sp., with its intermeshing microtrichia and a pair of rivet-like structures on the coxae, has a unique jumping mechanism. Among the four insects, *Tricentrus* sp. is notable for its opposable gears on the trochanters. However, these gears apparently do not contribute to hind leg synergy, leading to a tendency for *Tricentrus* sp. to tilt to one side during jumping. In the studied insects, the wings did not contribute to the energy required for jumping either at take-off or during the gliding phase. However, in *Kolla* sp., wing extension occurred 100 ms before take-off, with repeated wing beats culminating in an elevated position during take-off, although no wing flapping occurred during gliding. Leafhoppers’ preparatory movements for take-off include swaying their bodies from side to side, possibly allowing both hind legs to build up energy and adjust the jump direction. The irregular and unstable jumping of Auchenorrhyncha provides good adaptability and survival activity to avoid adverse environmental factors, such as natural enemies.

While direct observation of the distance and vertical height of jumps was not feasible in the studied insects. The use of a high-speed camera at a specific angle allowed for the tracking of individual leg movements, but using this method, it was not possible to capture the complete trajectory of the insects’ jumps. To overcome this limitation, standard equations for the motion of an inert body were employed [32] (Equations (1) and (2) below) to estimate the horizontal distance and vertical height achieved during the jump. These calculations were made without considering the contribution of the wings and under the assumption of negligible aerodynamic drag on the body:

(1) s=vcos⁡θ(2vsin⁡θ/9.81),

(2) $${h=(v\sin\theta )}^{2}/(2\times 9.81),$$

where *s* is the distance jumped, *h* is the maximum height reached, *v* is the instantaneous velocity at take-off, *θ* is the take-off angle (Table 2) and g is the acceleration due to gravity (9.81 ${ms}^{-2}$).

The jumping motion of insects is not a standard curvilinear trajectory. The horizontal distances and vertical heights we calculated are theoretical estimates based on the simplification of the insect as a particle and the assumption of negligible aerodynamic drag [33,34]. These calculations suggest that these insects can cover remarkable distances, given their small body sizes (5.2 to 7.6 mm): *Kolla* sp. can leap to over 1.3 m, *Euricania ocellus* to nearly 0.8 m, *Lepyronia coleoptrata* to about 0.4 m and *Tricentrus* sp. to almost 0.5 m (Table 3). Vogel’s research suggested that the actual jumping range can be significantly reduced due to drag; for example, up to 25% in the froghopper *Philaenus spumarius* [34]. Smaller insects, such as flea beetles, may lose up to 40% of their jumping capabilities, and even smaller fleas (Siphonaptera) could lose as much as 80% of their jumping capabilities due to drag forces [32]. Despite these potential losses, the rapid acceleration, take-off velocity, and the projected distance of the upward and forward jumps of the studied species rank these insects among the most proficient leapers on par with the previously studied spittlebug, *Philaenus spumarius* [8].

## 5. Conclusions

In summary, the present results highlight that while the jumping principle remained consistent across the four insect families studied, the ultrastructural variations in their hind legs resulted in significant differences in the jumping mechanisms. Specifically, a higher degree of intermeshing in the coxa–trochanter correlated with increased jumping stability. Furthermore, the meshing degree of the coxa was superior to that of the trochanter.

## Figures and Tables

**Table 1:** Body length and mass, and lengths of the hind leg femora and tibiae in four species.

	Body Mass (mg)	Body Length (mm)	Femur (mm)	Tibia (mm)	Hind Leg Length (% Body Length)
*Lepyronia coleoptrata* (N = 17)	18.6 ± 3.4	7.6 ± 0.3	1.3 ± 0.1	3.1 ± 0.1	84
*Euricania ocellus* (N = 13)	11.8 ± 0.3	6.1 ± 0.05	1.5 ± 0.1	2.2 ± 0.1	86
*Kolla* sp. (N = 14)	3.9 ± 0.4	6.7 ± 0.1	1.4 ± 0.05	2.4 ± 0.1	88
*Tricentrus* sp. (N = 9)	8.2	5.2	0.7	2.4	85

N, the number of insects analyzed in this table.

**Table 2:** The jumping performance of the four species.

	Body Mass (mg)	Time to Take-Off (ms)	Take-Off Velocity $\left(ms^{-1}\right)$	Take-Off Angle (deg)	Acceleration $(ms^{-2}$)	g-Force (g)	Energy (μJ)	Power (mW)	Force (mN)
Formula/symbol	*m*		*v*		*f* = *v*/*t*	*g* = *f*/9.81	*e* = 0.5 m$v^{2}$	*P* = *e*/*t*	*F* = *mf*
*Lepyronia coleoptrata*			2						
mean (N = 17, n = 3)	18.6 ± 3.4	1.7 ± 0.1		37 ± 4	1118	114	33	19	21
best	19.1	1.6			1250	127	38	23	24
*Euricania ocellus*			2.8						
mean (N = 13, n = 7)	11.8 ± 0.3	1.3 ± 0.1		48 ± 2	2077	212	43	33	25
best	11.6	1.2			2333	238	45	38	27
*Kolla* sp.			3.9						
mean (N = 14, n = 2)	3.9 ± 0.4	4.2 ± 0.2		30 ± 6	857	87	25	6	3
best	4.1	4			975	99.	38	10	4
*Tricentrus* sp.									
mean (N = 9, n = 1)									
best	8.2	1.7	2.4	29	1412	144	24	14	12

The values in the five columns on the right were calculated from the mean values given in the four columns on the left. The best performance of a particular individual for each species is also given. Only data for the jumps that were viewed from the side are presented here. N, the number of insects analyzed in this table; n, the number of jumps.

**Table 3:** The jumping distances and heights of the four species.

Species	Take-Off Velocity $\left(ms^{-1}\right)$	Take-Off Angle (deg)	Body Length (mm)	Horizontal Distance (mm)	Horizontal Distance (Body Lengths)	Vertical Height (mm)	Vertical Height (Body Lengths)
*Lepyronia coleoptrata*	2	37	7.6	392	52	74	10
*Euricania ocellus*	2.8	48	6.1	795	130	221	36
*Kolla* sp.	3.9	30	6.7	1343	200	737	110
*Tricentrus* sp.	2.4	29	5.2	498	96	93	18

## Data Availability

Most data analyzed and modeled during this study are included in this published article. The rest are available from the corresponding author upon reasonable request.
